# Effects of Granulocyte-Macrophage Colony-Stimulating (GM-CSF) Factor on Corneal Epithelial Cells in Corneal Wound Healing Model

**DOI:** 10.1371/journal.pone.0138020

**Published:** 2015-09-16

**Authors:** Chang Rae Rho, Mi-young Park, Seungbum Kang

**Affiliations:** 1 Department of Ophthalmology and Visual Science, Daejeon St. Mary's Hospital, College of Medicine, The Catholic University of Korea, Seoul, Republic of Korea; 2 Clinical Research Institute, Daejeon St. Mary's Hospital, College of Medicine, The Catholic University of Korea, Daejeon, Republic of Korea; Cedars-Sinai Medical Center, UNITED STATES

## Abstract

Granulocyte-macrophage colony-stimulating factor (GM-CSF) is a pleiotropic cytokine that activates granulocyte and macrophage cell lineages. It is also known to have an important function in wound healing. This study investigated the effect of GM-CSF in wound healing of human corneal epithelial cells (HCECs). We used human GM-CSF derived from rice cells (rice cell-derived recombinant human GM-CSF; rhGM-CSF). An in vitro migration assay was performed to investigate the migration rate of HCECs treated with various concentrations of rhGM-CSF (0.1, 1.0, and 10.0 μg/ml). MTT assay and flow cytometric analysis were used to evaluate the proliferative effect of rhGM-CSF. The protein level of p38MAPK was analyzed by western blotting. For in vivo analysis, 100 golden Syrian hamsters were divided into four groups, and their corneas were de-epithelialized with alcohol and a blade. The experimental groups were treated with 10, 20, or 50 μg/ml rhGM-CSF four times daily, and the control group was treated with phosphate-buffered saline. The corneal wound-healing rate was evaluated by fluorescein staining at the initial wounding and 12, 24, 36, and 48 hours after epithelial debridement. rhGM-CSF accelerated corneal epithelial wound healing both in vitro and in vivo. MTT assay and flow cytometric analysis revealed that rhGM-CSF treatment had no effects on HCEC proliferation. Western blot analysis demonstrated that the expression level of phosphorylated p38MAPK increased with rhGM-CSF treatment. These findings indicate that rhGM-CSF enhances corneal wound healing by accelerating cell migration.

## Introduction

An intact corneal epithelium is necessary to maintain corneal transparency and protect against infection. Stromal ulcers can arise from persistent epithelial defects, leading to decreased visual acuity [1]. Controlled and early epithelial wound healing is very important for epithelial defects induced by clean wounds such as those associated with photorefractive keratectomy and epithelial laser in situ keratomileusis as well as for those induced by pathologic wounds including neurotrophic ulcers, chemical burns, and infection. This healing process is influenced by growth factors that coordinate cell migration, adhesion, and proliferation processes. Known growth factors include epidermal growth factor, hepatocyte growth factor, keratinocyte growth factor, platelet-derived growth factor, nerve growth factor, and insulin-like growth factor. These are released from the corneal stroma and lacrimal glands [2–5].

Granulocyte-macrophage colony-stimulating factor (GM-CSF) is a pleiotropic cytokine that activates granulocyte and macrophage cell lineages [6]. It is also known to have an important function in wound healing. In vivo studies using GM-CSF have shown that it positively affects wound healing by inducing keratinocyte proliferation and promoting migration of epithelial cells [7]. Some studies have demonstrated considerable effects of GM-CSF on the healing of cuts, burns, leg ulcers, and skin grafts [8].

In the present study, we investigated the effect of rice cell-derived recombinant human GM-CSF (rhGM-CSF) on wound healing. Protein products obtained from plants are safer and more cost-effective than those obtained using traditional systems such as microbial cultures, animal cell cultures, or transgenic animals [9]. The effect of this exogenous protein on corneal wound healing is unknown.

The purpose of the present study was to determine the influence of topical rhGM-CSF on wound healing in a hamster corneal epithelial wound model and investigate the mechanism of the effect of rhGM-CSF.

## Material and Methods

### Cell culture and experimental animals

Human corneal epithelial cells (HCECs) were purchased from the American Type Culture Collection (SV40-immortalized HCEC line; Rockville, MD) and cultured in Dulbecco’s Modified Eagle’s Medium/Nutrient Mixture F-12 (Gibco-Life Technologies, Carlsbad, CA) supplemented with 5% fetal bovine serum (Gibco-Life Technologies), 5 μg/ml insulin (Sigma, St. Louis, MO), penicillin/streptomycin (1:100), 500 ng/ml hydrocortisone (Sigma), 30 ng/ml cholera toxin (Sigma), and 10 ng/ml human epithelial growth factor (Sigma) in a 37°C incubator under a humidified atmosphere containing 5% CO_2_. In total, 100 male golden Syrian hamsters (age, 10 weeks; weight, 120–140 g) were used in the study. All animal studies were approved by the Institutional Animal Care and Use Committee of the College of Medicine, Catholic University of Korea (Permit Number: CMCDJ-AP-2013-009). All hamsters were handled in accordance with the Association for Research in Vision and Ophthalmology (ARVO) Statement for the Use of Animals in Ophthalmic and Vision Research. All surgery was performed under general anesthesia, and all efforts were made to minimize suffering. rhGM-CSF was donated from the manufacturer (CHA Bio & Diostech Inc., Seoul, Korea). Various concentrations of rhGM-CSF were used to treat the cultured cells or were topically applied to the eyes of the hamsters.

### *In vitro* migration assay

An in vitro migration assay to investigate the migration rate of HCECs was performed using ibidi Culture-Inserts (ibidi GmbH, Martinsried, Germany) according to the manufacturer’s instructions. HCECs were seeded into the Culture-Inserts and grown to confluency. Growth factor-starved HCECs were treated with various concentrations of rhGM-CSF (0.1, 1.0, and 10.0 μg/ml). The Culture-Inserts within the cell plates were then removed to create a linear scratch wound. Images of the scratch wound fields were captured with an inverted light microscope (Eclipse TE300; Nikon, Tokyo, Japan) equipped with a digital camera at 0, 24, and 48 h of incubation. HCECs were stained with tetramethylrhodamine isothiocyanate (TRITC)-conjugated phalloidin (Life technologies, Carlsbad, CA). The migration rate was defined as the ratio of the difference between the initial wound area and the remaining wound area vs. the initial wound area. The remaining wound areas photographed at 24 and 48 h were quantified using ImageJ software (National Institutes of Health, Bethesda, MD). Experiments were performed at least three times.

### *In vivo* corneal epithelial wound healing

Golden Syrian hamsters (Central Lab Animal Inc., Seoul, Korea) were anesthetized with intraperitoneal injection of 30 mg of zolazepam (Zoletil; Virbac, Carros, France) and 10 mg of xylazine hydrochloride (Rompun; Bayer, Leverkusen, Germany) per kilogram of body weight and one drop of 0.5% proparacaine hydrochloride (Alcaine; Alcon, Fort Worth, TX) in each eye. Circular, 3-mm central corneal epithelial defects were created in only one eye of each animal. Briefly, a 3-mm trephine was placed on the cornea to demarcate the central corneal region of the eye. After 30 s of alcohol delamination of the central corneal epithelium, the epithelium was scraped and removed using a spatula and microblade under a microscope. One one drop of 0.5% proparacaine hydrochloride was instilled in the eye postoperatively to minimize suffering. Based on the Resource Equation method, we made four groups with 6 hamsters each. The eyes of the hamsters in each of the four groups were treated with phosphate-buffered saline (PBS) (control group) or 10, 20, or 50 μg/ml of rhGM-CSF solution. Levofloxacin eye drop (Oculevo, Samil, Seoul, Korea) was given four times daily at equal intervals until the healing of the corneal epithelial defects was complete. The in vivo experiment was repeated three more times.

### Quantification of corneal epithelial wound healing

Each eye was stained with one drop of 1% fluorescein and then irrigated with PBS. The wound size was photographed at 12-h intervals to determine the rate of corneal wound healing. The area of the fluorescein-stained epithelial defect was quantified using ImageJ software (National Institutes of Health). The corneal wound healing data were analyzed as the percentage of wound remaining, which was calculated as follows: fluorescein-stained epithelial defect area divided by initial fluorescein-stained defect area.

### MTT assay

A 3-(4,5-dimethylthiazol-2-yl)-2,5-diphenyl tetrazolium bromide (MTT) assay, which serves as an indirect marker for cell proliferation and cytotoxicity, was used to verify the effect of rhGM-CSF on the proliferation of HCECs. The HCECs (1 × 10^4^ cells/well) were plated in a 96-well plate. After 12 h, the cells were treated with different concentrations of rhGM-CSF (0.1, 1.0, and 10.0 μg/ml) and further incubated for 48 h. The cells were then incubated with 10 μl of MTT solution for 4 h at 37°C. The absorbance was measured at 450 nm by a microplate reader (model 680; Bio-Rad, Hercules, CA). The same experiment was repeated in three separate cultures. The absorbance values were expressed as a percentage of the control condition representing 100% cell proliferation.

### Flow cytometric measurement of HCEC proliferation

The proliferation of HCECs was assessed by analyzing the cell cycle. HCECs were seeded in the plate and allowed to attach for 24 h. They were then treated with varying concentrations of rhGM-CSF (0.1, 1.0, and 10.0 μg/ml) and incubated for another 24h. For the flow cytometry assays, HCECs were detached with trypsin-EDTA, and the pellet was resuspended in 70% ethanol solution and stained with propidium iodide (Sigma). HCECs treated with rhGM-CSF were analyzed by flow cytometry (BD FACSCanto II; BD Biosciences, San Jose, CA). Ten thousand cells were counted per sample, and data were processed with BD FACSDiva software (BD Biosciences). The results are expressed as the percentage of cells in the S phase of the cell cycle.

### Western blot analysis

A standard western blotting method was used. Briefly, HCECs were serum-starved overnight and stimulated with different concentrations of rhGM-CSF (0.1, 1.0 and 10.0 μg/ml). After 60 min, the HCECs were harvested and pooled in 100 μl of lysis buffer (Pro-prep Protein Extraction Solution; iNtRON Biotechnology, Seongnam, Korea). Cell lysates were centrifuged at 13,000 rpm for 15 min at 4°C, and the supernatants were collected. After measuring the protein concentration (BCA Protein Assay Kit; Pierce, Rockford, IL), equal amounts of protein were separated by electrophoresis on a 10% SDS-polyacrylamide gel and transferred electrophoretically onto a nitrocellulose membrane (Bio-Rad). After blocking with a 3% BSA solution, the membrane was incubated overnight at 4°C with an anti-phospho-p38 mitogen-activated protein kinase (MAPK) antibody (Cell Signaling, Danvers, MA) and an anti-p38 MAPK antibody (Cell Signaling) to detect phosphorylated p38 MAPK and total p38 MAPK, respectively. β-actin served as an internal control. The intensity of the signals was recorded and quantified by a molecular imaging system (Molecular Imager ChemiDoc XRS+; Bio-Rad). The expression levels of phosphorylated p38 MAPK were normalized to those of total p38 MAPK.

### Statistical analysis

Unless indicated otherwise, data are expressed as means ± standard deviation. The significance of differences between groups was evaluated using ANOVA or the Mann–Whitney U test. A P value of <0.05 was considered to indicate statistical significance. SPSS version 17.0 (SPSS, Chicago, IL) was used for statistical analysis.

## Results

### Accelerated migration of HCECs by rhGM-CSF

**Fig 1:**
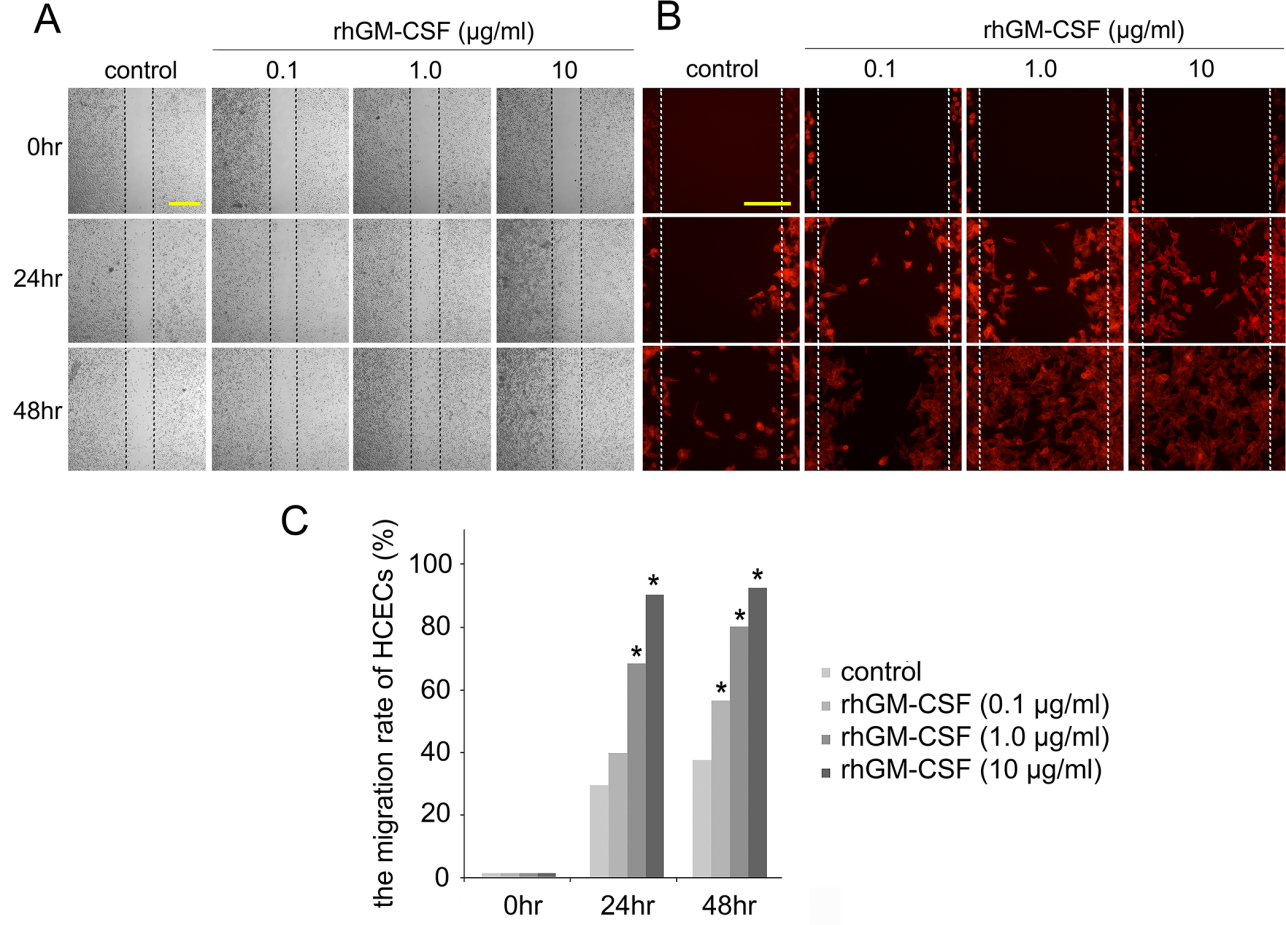
In vitro migration assay. (A) rhGM-CSF promotes healing of scratch wounds in HCECs. Scratch wounds were made on plates of confluent HCECs and allowed to heal in medium containing 0.1, 1.0, and 10.0 μg/ml rhGM-CSF. The wounded layers were incubated for 48 h. Wound closure was photographed at 0, 24, and 48 h after wounding. Three independent experiments were performed. Scale bar, 500㎛. (B) F-actin staining with phalloidin (red) demonstrates the morphology of migrating HCECs. Scale bar, 200㎛. (C) The migration rate of HCECs was increased by rhGM-CSF treatment in a dose-dependent manner. Plotted data are the mean migration rates of HCECs at 0, 24, and 48 h. The migration rate was defined as the percentage of resurfaced area divided by the initial wound area. *P < 0.05 vs. the migration rate of the control.

Wound healing in the in vitro scratch assay was evaluated to determine the role of rhGM-CSF on epithelial wound closure. Our findings showed that the migration rates of HCECs were significantly enhanced as the concentration of rhGM-CSF increased (Fig 1A). In addition, phalloidin-stained HCECs in high magnification showed that higher rhGM-CSF concentrations are associated with more evident morphologic changes of HCECs (Fig 1B). The basal wound-healing rates of HCECs were 29.1% ± 8.4% at 24 h and 37.2% ± 10.8% at 48 h. At 24 h post-wounding, the corneal epithelial wound-healing rates in the 0.1, 1.0, and 10.0 μg/ml rhGM-CSF-treated cells were 40.1% ± 11.4%, 69.7% ± 13.8%, and 92.0% ± 4.9%, respectively. At 48 h post-wounding, the corneal epithelial wound-healing rates in the 0.1, 1.0, and 10.0 μg/ml rhGM-CSF-treated cells were 56.7% ± 11.9%, 82.0% ± 19.1%, and 93.5% ± 3.2%, respectively. Statistical significance was noted in the 1.0 and 10.0 μg/ml rhGM-CSF-treated HCECs at 24 h post-wounding (P < 0.05). Statistical significance was also noted in all rhGM-CSF-treated cells at 48 h post-wounding (P < 0.05). rhGM-CSF significantly promoted the migration rate of HCECs and shortened the epithelial wound closure time compared with the control (Fig 1C).

### Promotion of corneal epithelial wound healing by rhGM-CSF

**Fig 2:**
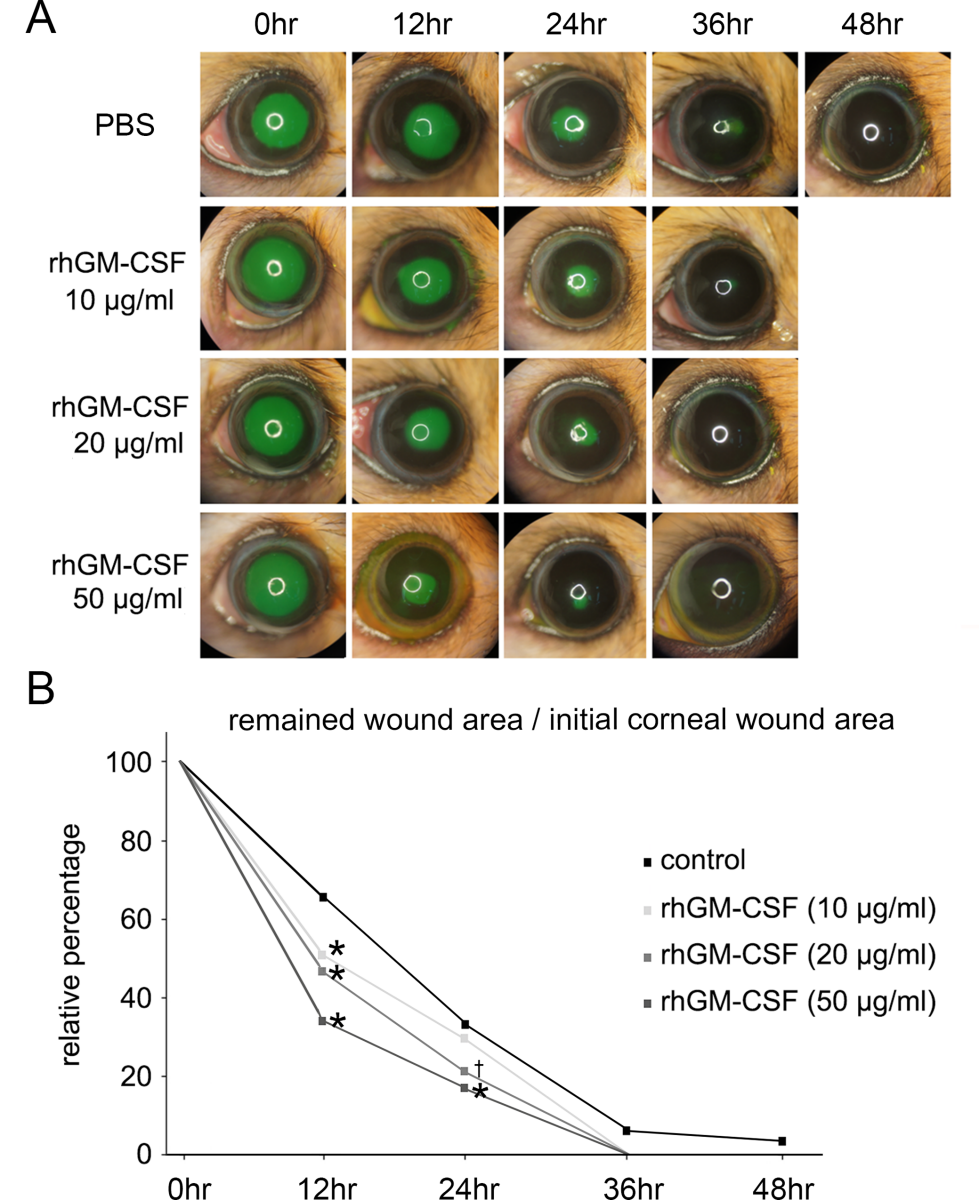
*In vivo* corneal epithelial wound healing. (A) rhGM-CSF promoted accelerated corneal wound healing. Slit lamp photographs showed fluorescein staining of corneal epithelial wounds in the control and rhGM-CSF (10, 20, and 50 μg/ml) treatment groups at 0, 12, 24, 36, and 48 h after debridement. (B) Plotted data are the relative percentages of the corneal wound area at 12, 24, 36, and 48 h. The area of the initial corneal epithelial wound was normalized to 100%. The wound-healing rate of rhGM-CSF-treated eyes was much faster than that of untreated eyes. *P < 0.01 vs. control; †P = 0.02 vs. control.

In the control eyes without rhGM-CSF treatment, most corneal epithelial wounds were resurfaced within 48 h. In the rhGM-CSF-treated eyes, however, all corneal epithelial wounds were completely healed within 36 h at all concentrations tested (Fig 2A). Quantitative analysis revealed that the denuded areas in the rhGM-CSF-treated eyes (10, 20, and 50 μg/ml) were significantly smaller than those in the control eyes at 12 h after epithelial debridement (P < 0.01). At 24 h, the defect areas in the rhGM-CSF-treated eyes (20 and 50 μg/ml) were significantly smaller than those in the control eyes (20 μg/ml rhGM-CSF vs. control, P = 0.02; 50 μg/ml rhGM-CSF vs. control, P < 0.01) (Fig 2B). In the control and rhGM-CSF-treated eyes, the epithelial defects were resurfaced without any haze or opacity during the healing period.

### No effects of rhGM-CSF on proliferative activity of HCECs

**Fig 3:**
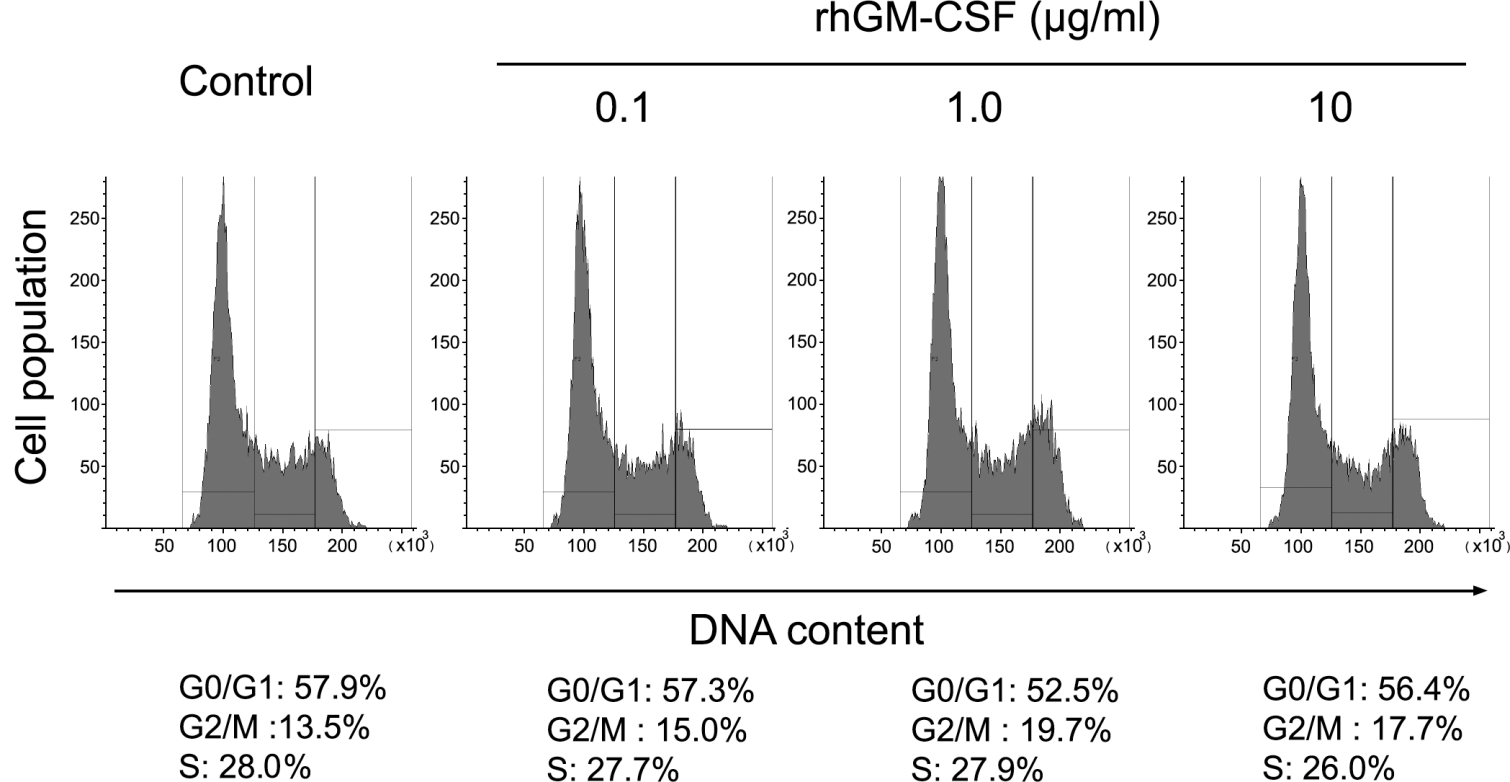
Flow cytometry analysis of rhGM-CSF effect on HCECs. HCEC proliferation was quantified by S-phase fraction measurement by flow cytometry. HCECs were treated with 0.1, 1.0, and 10.0 μg/ml rhGM-CSF, and flow cytometric measurements of the S-phase fraction were then performed. The data show the percentages of the cells in the S phase of the cell cycle.

Growth factors are usually involved in cell migration and proliferation. Therefore, MTT assay and cell-cycle analysis using flow cytometry were carried out at various concentrations of rhGM-CSF (0.1, 1.0, and 10.0 μg/ml) to evaluate the effects of rhGM-CSF on proliferation of HCECs. The MTT assay demonstrated that rhGM-CSF treatment up to 10 μg/ml exhibited no effects on HCEC proliferation (S1 Fig). The respective percentages of proliferation are 100% in the control, 101.5% in 0.1 μg/ml, 105.6% in 1.0 μg/ml, and 102.9% in 10 μg/ml rhGM-CSF-treated HCECs. Flow cytometry was used to evaluate whether rhGM-CSF is capable of increasing cell proliferative activity, defined as the S-phase fraction of cycling cells. In rhGM-CSF-untreated HCECs, the percentages of cells in the G0/G1, G2/M, and S phase were 57.9%, 13.5%, and 28.0%, respectively. The percentages of 0.1, 1.0, and 10.0 μg/ml rhGM-CSF-treated cells in the G0/G1 phase were 57.3%, 52.5%, and 56.4%; those in the G2/M phase were 15.0%, 19.7%, and 17.7%, and those in the S phase were 27.7%, 27.9%, and 26.0%, respectively. rhGM-CSF (0.1, 1.0, and 10.0 μg/ml) did not alter the S phase population at 24 h, suggesting that rhGM-CSF does not promote HCEC proliferation (Fig 3).

### Activation of p38 MAPK by rhGM-CSF

**Fig 4:**
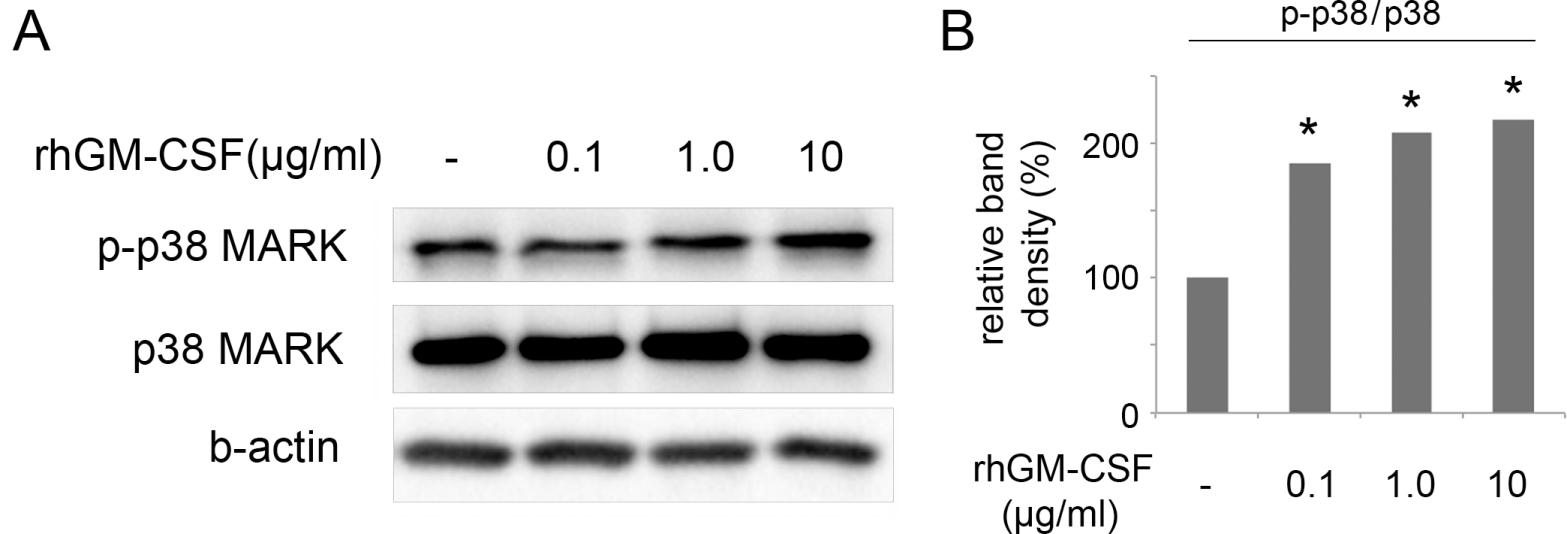
Evaluation of p38 MAPK signaling pathway by western blotting. (A) rhGM-CSF induced activation of the p38 MAPK signaling pathway. HCECs were treated with different concentrations of rhGM-CSF (0.1, 1.0, and 10.0 μg/ml) for 60 min. The expression of phosphorylated and total p38 MAPK was then analyzed by western blotting. (B) The expression levels of phosphorylated p38 MAPK were normalized to those of total p38 MAPK, which increased with rhGM-CSF treatment. Three independent experiments were performed. *P < 0.05 vs. control.

Activation of MAPKs, including Jun N-terminus kinase, p38, and ERK, is necessary for migration. Among the MAPKs, p38 MAPK is known to play crucial roles in cell migration, especially in terms of its directionality. Therefore, we investigated whether rhGM-CSF treatment can activate p38 MAPK phosphorylation in HCECs. Western blot analysis demonstrated that the expression level of phosphorylated p38 MAPK increased with rhGM-CSF treatment at all concentrations tested (Fig 4A). The expression levels of phosphorylated p38 MAPK relative to those of total p38 MARK in the 0.1, 1.0, and 10.0 μg/ml-treated HCECs were 1.83-, 2.14-, and 2.32-fold greater than those in the non-treated HCECs, respectively. Significant differences were noted in all rhGM-CSF-treated samples (P < 0.05) (Fig 4B).

## Discussion

Early corneal wound healing is essential to maintain corneal transparency and a barrier to the external environment. Delayed epithelial healing in neurotrophic keratopathy can progress to a persistent epithelial defect, potentially leading to stromal scarring, neovascularization, perforation, and secondary bacterial infection.

Several growth factors and related membrane receptors are involved in cell migration and attachment. These growth factors have been suggested to control the healing of the corneal epithelial wound [10–12]. An earlier study showed that epidermal growth factor stimulates corneal epithelial cell attachment to fibronectin [13]. Another experiment suggested that insulin-like growth factor promotes corneal epithelial cell migration and laminin-5 production [14]. New therapies to modulate the epithelialization in corneal wound healing include 17β-estradiol, nicergoline, and β-glucan [1].

Various reports have addressed the beneficial effect of GM-CSF for skin wounds and oral mucositis [15–17]. In one study, cutaneous wound healing was significantly delayed in GM-CSF-knockout mice, suggesting the important role of GM-CSF in the wound healing process [18]. In the present work, we evaluated the potential positive effects of rhGM-CSF in a corneal epithelial wound model. Our results showed that rhGM-CSF accelerated corneal epithelial wound healing both in vitro and in vivo. The enhancement of wound healing was dependent on the rhGM-CSF concentration. The healing rate increased as the concentration of the rhGM-CSF increased. We examined the proliferation of epithelial cells to elucidate the mechanism of rhGM-CSF in wound healing enhancement. MTT assay and cell cycle analysis using flow cytometry showed no significant differences with rhGM-CSF treatment. This implicates that rhGM-CSF does not activate corneal epithelial cell proliferation. Rather, the underlying mechanism in the acceleration was attributed to increased cellular migration based on the p38 MAPK western blot results. Saika et al. demonstrated that p38 MAPK plays a major role in the acceleration of cell migration and in suppression of cell proliferation in migrating mouse corneal epithelium [19].

A recent study investigated the effects of stem cell factor (SCF) and c-kit in corneal wound healing in mice [20]. In vivo and in vitro experiments indicated that the SCF/c-kit system is most likely implicated in cell migration and cell attachment, not cell proliferation, during corneal epithelial wound healing [20]. These results are in accordance with those obtained in the present study. SCF, also known as mast cell growth factor, synergizes with GM-CSF, and both factors share components of signaling pathways [21]. Although we did not find a specific receptor for GM-CSF in the corneal epithelium, GM-CSF may activate corneal epithelial cell migration via the c-kit system.

Although the detection rate of GM-CSF in tears varies among reports, GM-CSF is reportedly present in tears. A recent analysis of tears from normal subjects revealed that GM-CSF was detected in 16 of 18 eyes at a mean level of 28.4 ± 4.0 pg/ml [22]. However, La France et al. detected GM-CSF in the tears of only 30% of their subjects [23]. Because GM-CSF can be found in normal tears, it may be able to control the interaction of cell adhesion molecules with extracellular matrices during epithelial wound healing, similar to other growth factors [14, 24]. However, we found no specific adhesion molecules related to GM-CSF. Although Miyamoto et al. attempted to identify adhesion molecules regulated by SCF, none of the molecules changed significantly [20]. However, they suggested that the SCF/c-kit system might be involved in cell attachment through enhancement of the avidity or affinity of integrins to fibronectin, laminin, and type IV collagen. Thus, growth factors may be able to change the functional state of integrins from an inactive state without changing the integrin expression [25].

In the present experiment, we evaluated the effect of rice cell-derived rhGM-CSF on corneal wound healing. There are a few known advantages associated with producing medical agents from plant cells [26–28]. First, the medium with which to maintain plant cells costs less than that needed to maintain animal cells. Second, fewer protein ingredients are incorporated in plant culture medium than in animal culture medium. Thus, it is more straightforward to refine the target protein from the medium. Third, rice cell-derived GM-CSF has a twofold higher glycosylation ratio than does yeast-derived GM-CSF. Previous experiments have shown that rice cell-derived GM-CSF is more stable than commercial yeast-derived GM-CSF in vivo [29].

One possible concern of using GM-CSF is that GM-CSF is known to attract inflammatory cells. However, we observed no increase in the infiltration of inflammatory cells in the corneas treated with rhGM-CSF. Again, no abnormal angiogenesis was found in the corneas treated with rhGM-CSF.

Previous literatures showed the beneficial effects of GM-CSF in chronic ulcerated skin, pressure ulcer, and delayed wound healing by providing more favorable environment [7, 30]. Therefore, we believe that GM-CSF could be also applied for various ocular diseases associated with delayed wound healing, including persistent epithelial defect of cornea, chronic superficial punctate keratopathy, and corneal epithelial defect due to limbal cell dysfuction.

In conclusion, topically applied plant-derived rhGM-CSF increased the wound-healing rate in vivo. This result indicates the possibility of developing rhGM-CSF as a treatment option for corneal epithelial wounds.

## Supporting Information

S1 Dataset Data for Figs 1–3 and S1 Fig. (XLSX)

S1 Fig MTT assay of rhGM-CSF effect on HCECs. (TIF)
