# Supplementary figures and images for: Effects of Granulocyte-Macrophage Colony-Stimulating (GM-CSF) Factor on Corneal Epithelial Cells in Corneal Wound Healing Model
Source: PLoS One. 2015 Sep 16;10(9):e0138020. doi: 10.1371/journal.pone.0138020 (PMC4574106; doi:10.1371/journal.pone.0138020)

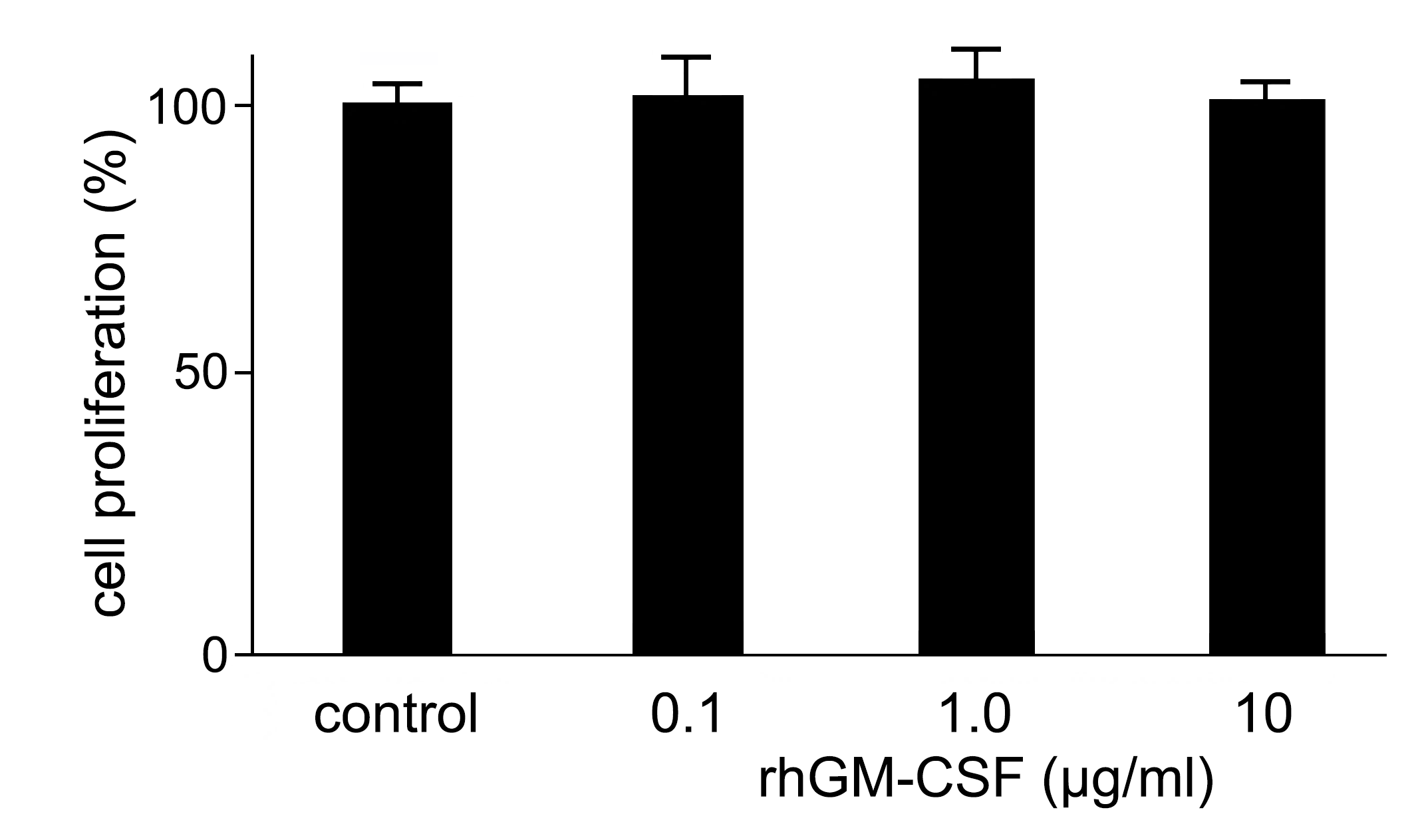

Supplement: S1 Fig — (TIF) [file pone.0138020.s002.tif]
